# Health profile of pediatric Special Immigrant Visa holders arriving from Iraq and Afghanistan to the United States, 2009–2017: A cross-sectional analysis

**DOI:** 10.1371/journal.pmed.1003069

**Published:** 2020-03-17

**Authors:** Simone S. Wien, Gayathri S. Kumar, Oleg O. Bilukha, Walid Slim, Heather M. Burke, Emily S. Jentes

**Affiliations:** 1 Immigrant, Refugee, and Migrant Health Branch, Division of Global Migration and Quarantine, Centers for Disease Control and Prevention, Atlanta, Georgia, United States of America; 2 Oak Ridge Institute for Science and Education, Oak Ridge, Tennessee, United States of America; 3 Emergency Response and Recovery Branch, Division of Global Health Protection, Centers for Disease Control and Prevention, Atlanta, Georgia, United States of America; 4 Migration Health Division, International Organization for Migration, Erbil, Iraq; 5 Migration Health Division, International Organization for Migration, Amman, Jordan; Johns Hopkins University Bloomberg School of Public Health, UNITED STATES

## Abstract

**Background:**

The United States has admitted over 80,000 Special Immigrant Visa holders (SIVH), which include children. Despite the increase in the proportion of SIVH admissions to the US over recent years, little is known about health conditions in SIV children. We report the frequency of selected diseases identified overseas and assess differences in selected conditions between SIV children from Iraq and Afghanistan.

**Methods and findings:**

We analyzed 15,729 overseas medical exam data in Centers for Disease Control and Prevention’s Electronic Disease Notification system (EDN) for children less than 18 years of age from Iraq (29.1%) and Afghanistan (70.9%) who were admitted to the US from April 2009 through December 2017 in a cross-sectional analysis. Variables included age, sex, native language, measured height and weight, and results of the overseas medical examination. From our analysis, less than 1% of SIV children (Iraqi: 0.1%; Afghan: 0.12%) were reported to have abnormal tuberculosis test findings, less than 1% (Iraqi: 0.3%; Afghan: 0.7%) had hearing abnormalities, and about 4% (Iraqi: 6.0% Afghan: 2.9%) had vision abnormalities, with a greater prevalence of vision abnormalities noted in Iraqis (OR: 1.9, 95% CI 1.6–2.2, *p* <0.001). Seizure disorders were noted in 46 (0.3%) children, with Iraqis more likely to have a seizure disorder (OR: 7.6, 95% CI 3.8–15.0, *p* < 0.001). On average, children from Afghanistan had a lower mean height-for-age z-score (Iraqi: −0.28; Afghan: −0.68). Only the data quality assessment for height for age for children ≥5 years fell within WHO recommendations. Limitations included the inability to obtain all SIVH records and self-reported medical history of noncommunicable diseases.

**Conclusion:**

In this investigation, we found that less than 1% of SIV children were reported to have abnormal tuberculosis test findings and 4% of SIV children had reported vision abnormalities. Domestic providers caring for SIVH should follow the US Centers for Disease Control and Prevention (CDC) Guidelines for the US Domestic Medical Examination for Newly Arriving Refugees, including an evaluation for malnutrition. Measurement techniques and anthropometric equipment used in panel site clinics should be assessed, and additional training in measurement techniques should be considered. Future analyses could further explore the health of SIV children after resettlement in the US.

## Abstract 2

Emily Jentes and coauthors report on the health of children from Iraq and Afghanistan in the United States.

## Author summary

**Why was this analysis done?:**

- Special Immigrant Visa holders (SIVH) include Iraqi or Afghan nationals who served the US government or military in Iraq or Afghanistan.
- SIVH and their families are eligible for refugee health benefits in the US, including a medical screening exam within 90 days of arrival. While SIVH have been entering the US in increasing numbers, little is known about the health of this population to guide public health practitioners and clinicians who provide care for these populations in the US.

**What did the researchers do and find?:**

- We analyzed over 15,700 medical records of SIV children who entered the US from 2009 to 2017 for select diseases and conditions.
- We found that less than 1% of SIV children had abnormal tuberculosis test results, and 4% of SIV children had vision abnormalities.
- On average, children from Afghanistan had a lower mean height-for-age z-score.
- The poor data quality of height and weight measurements for children under five years of age made it difficult to make conclusions about nutrition status.

**What do these findings mean?:**

- Domestic providers caring for SIV children should follow CDC’s Guidelines for the US Domestic Medical Examination for Newly Arriving Refugees, including a thorough evaluation for latent tuberculosis and malnutrition.
- CDC is currently working with overseas partners to assess height and weight measurement techniques and quality of measurement equipment to help more accurately determine the nutritional status of SIV children.

## Introduction

The US has admitted over 80,000 Special Immigrant Visa holders (SIVH). SIVH include people who worked with the US government as a translator or interpreter in Iraq or Afghanistan or in another capacity, as well as their dependents [1]. Unlike other immigrants, SIVH may elect to receive refugee benefits and services; therefore, they are processed similarly to US-bound refugees. Per the Immigration and Nationality Act, SIVH are required to undergo an overseas medical examination performed by a panel physician designated by the US Department of State. The US Centers for Disease Control and Prevention (CDC) writes the Technical Instructions for the Medical Examination of Aliens (referred to as the “Technical Instructions”) detailing how the exam is performed. Panel physicians are required to evaluate for certain inadmissible conditions (infectious tuberculosis, syphilis, gonorrhea, Hansen’s disease, physical or mental health disorders associated with harmful behavior [e.g., driving while intoxicated], and substance use disorders) but also document other medical conditions, which may be admissible, noted during the medical examination [2]. These admissible conditions are noted on the overseas medical exam as being absent or present, with a text section available for further remarks, if the physician indicates the condition is present [3].

The results of the examination are sent to US state and local health departments via CDC’s Electronic Disease Notification system (EDN) [4]. EDN is a centralized reporting system that notifies US state and local health departments and screening clinics of the arrival of all refugees, as well as immigrants with health conditions requiring medical follow-up, such as tuberculosis-related conditions. A copy of health records may be also collected upon arrival at US airports by CDC quarantine station staff and data sent to EDN; these can include health records from SIVH who may not have had health conditions requiring medical follow-up identified overseas.

Despite the increase in SIV admissions to the US over recent years, little is known about health conditions in SIV children. Increasing provider knowledge regarding common health conditions in SIV children may facilitate improved care upon arrival to the US. We report the frequency of selected diseases identified overseas and assess differences in selected conditions between SIV children from Iraq and Afghanistan.

## Methods

Iraqi and Afghan SIV children (<18 years) admitted to the US from April 2009 through December 2017 were included. EDN and the US Department of State’s Worldwide Refugee Admissions Processing System (WRAPS) were used as data sources in this cross-sectional analysis [1, 4]. We identified 15,729 SIV children <18 years of age.

EDN variables included age, sex, measured height and weight, and results of the overseas medical examination, while information about native language was obtained from WRAPS. Presence of noncommunicable diseases was self-reported and categorized as either “yes” or “no.”

This analysis was not guided by a specific prospective analysis plan. Frequencies were calculated to describe demographic characteristics and disease prevalence. Mean z-scores and standard deviations (SD) were calculated for weight for height (if <5 years), body mass index (BMI) for age (if ≥5 years), and height for age (all ages) using the World Health Organization’s Statistical Analysis Software macros [5, 6]. Data quality of these anthropometric measurements was assessed using cutoff points for SD according to World Health Organization’s recommendations [7]. Multivariable logistic regression models adjusted for age and sex were used to assess associations of certain conditions with nationality. Health conditions with fewer than 10 cases were excluded from regression analyses. Denominators used to estimate the prevalence of medical conditions varied because of missing data. All statistical analyses were performed with SAS 9.3 (SAS Institute, Cary, NC). This project was determined non-research by a CDC human subjects advisor; therefore, IRB review was not required. A STROBE Checklist (S1 STROBE Checklist) can be found under Supporting Information.

## Results

Our analysis included 15,729 children (Table 1). For children <5 years of age, the observed mean weight-for-height z-score in Afghan children was lower than in Iraqi children (Iraqi +0.13; Afghan: −0.10), as was observed mean height-for-age z-score (Iraqi: −1.09; Afghan: −1.37). However, the SDs for all anthropometric indicators were larger than the suggested range for data quality (Table 1), suggesting potential inaccuracy in measurement or reporting [7].

**Table 1:** Demographic characteristics and nutrition status of SIVH children less than 18 years resettling to the US, 2009–2017 (*n* = 15,729)^a^.

Demographic Characteristics	All *N* (%)	Iraq *N* (%)	Afghanistan *N* (%)
**Total**	15,729	4,579 (29.1%)	11,150 (70.9%)
**Sex**			
Female	7,488 (47.6%)	2,143 (46.8%)	5,345 (47.9%)
Male	8,241 (52.4%)	2,436 (53.2%)	5,805 (52.1%)
**Age**			
0–2 years	1,823 (11.6%)	382 (8.3%)	1,441 (12.9%)
3–5 years	6,152 (39.1%)	1,610 (35.2%)	4,542 (40.7%)
6 years and older	7,754 (49.3%)	2,587 (56.5%)	5,167 (46.3%)
**Native Language**	***N* = 15,471**	***N* = 4,478**	***N* = 10,993**
Dari	--	--	6,202 (56.4%)
Pashto	--	--	4,573 (41.6%)
Arabic	--	3,806 (85.0%)	--
Kurdi	--	563 (12.6%)	--
Others^b^	--	109 (2.4%)	218 (2.0%)
**Nutrition Status**^**c**^	**All**	**Iraq Mean z-Score (SD)**	**Afghanistan Mean z-Score (SD)**
**Ages 0 to <5 years**			
Weight for height (*n* = 7,853)	--	0.13 (1.64)	−0.10 (1.39)
Height for age (*n* = 7,865)	--	−1.09 (1.74)	−1.37 (1.56)
**Ages 5 to <18 years**			
Height for age (*n* = 7,778)	--	−0.28 (1.18)	−0.68 (1.20)
BMI for age (*n* = 7,681)	--	0.25 (1.54)	−0.41 (1.21)

^a^Percentages may not add up to 100% because of rounding.

^b^For native language, “Others” consisted of nine additional languages for Iraqi children, including Assyrian and Chaldean, and eight additional languages for Afghan children, including Turkmen and Uzbek.

^c^The World Health Organization states that SDs for z-scores greater than 1.3 are suggestive of inaccurate data, with the expected SD range of 1.10 to 1.30 for height-for-age z-score; 1.00 to 1.20 for weight-for-age z-score; and 0.85 to 1.10 for weight-for-height z-score.

Abbreviations: BMI, body mass index; SD, standard deviation; SIVH, Special Immigrant Visa holder

For children ≥5 years, the mean height-for-age z-score in Afghan children was lower than in Iraqi children (Iraqi: −0.28; Afghan: −0.68), as was mean BMI-for-age z-score (Iraqi: +0.25; Afghan: −0.41). However, the SDs for BMI for age were larger than expected (Table 1), suggesting potential inaccuracy in measurement or reporting [7].

Sixteen children had abnormal tuberculosis test findings: 3 (0.02%) had an abnormal chest X-ray with negative sputum cultures (Class B1) and, among those who received a tuberculin skin test (TST), 13 (0.1%) had a positive TST but negative chest X-ray (Class B2) (Table 2). Vision abnormalities were noted in 566 (4%) children, with Iraqis twice as likely to have vision abnormalities (OR: 1.9, 95% CI 1.6–2.2, *p* < 0.001). Among children reported with vision abnormalities, strabismus was reported for 92 children (0.5%). Hearing abnormalities were noted in 89 (0.6%) children, with Iraqis less likely to have hearing abnormalities (OR: 0.4, 95% CI 0.2–0.7, *p* = 0.002). Seizure disorders were noted in 46 (0.3%) children, with Iraqis more likely to have a seizure disorder (OR: 7.6, 95% CI 3.8–15.0, *p* < 0.001). No cases of syphilis, gonorrhea, or Hansen’s disease were found.

**Table 2:** Prevalence of admissible (Class B) tuberculosis (TB) conditions and other medical conditions among SIVH children less than 18 years of age resettling to the US, 2009–2017 (*n* = 15,729)^a^^,^^b^.

Medical Conditions	All *N* (%)	Iraq *N* (%)	Afghanistan *N* (%)	Adjusted Odds Ratio (95% Confidence Interval) Presence of Risk Factor/Health Condition	*p*-Value
**Class B TB Condition**					
No TB condition	15,709 (99.9%)	4,575 (99.9%)	11,134 (99.9%)	--	--
Class B1^c^	3 (0.02%)	1 (0.02%)	2 (0.02%)	--	--
Class B2^d^ (*n* = 12,874)	13 (0.1%)	3 (0.08%)	10 (0.1%)	--	--
**Medical Condition**^**e**^^,^^**f**^					
Illness or injury requiring hospitalization (*n* = 14,940)	813 (5.4%)	419 (9.7%)	394 (3.7%)	2.6 (2.2–2.9)	<0.001
Abnormal vision (*n* = 14,882)	566 (3.8%)	254 (6.0%)	312 (2.9%)	1.9 (1.6–2.2)	<0.001
Abnormal hearing (*n* = 14,881)	89 (0.6%)	12 (0.3%)	77 (0.7%)	0.4 (0.2–0.7)	0.002
Visible disabilities (*n* = 14,946)	82 (0.5%)	13 (0.3%)	69 (0.6%)	0.4 (0.2–0.9)	0.009
Impairment in learning, intelligence, or communication (*n* = 14,944)	67 (0.4%)	21 (0.5%)	46 (0.4%)	1.1 (0.6–1.8)	0.648
Seizure disorder (*n* = 14,946)	46 (0.3%)	35 (0.8%)	11 (0.1%)	7.6 (3.8–15.0)	<0.001
Major mental disorder (*n* = 14,944)	34 (0.2%)	12 (0.3%)	22 (0.2%)	1.3 (0.7–2.7)	0.40
Asthma (*n* = 14,945)	31 (0.2%)	27 (0.6%)	4 (0.04%)	NA	--
Congenital heart disease (*n* = 14,943)	28 (0.2%)	7 (0.2%)	21 (0.2%)	NA	--

^a^Percentages may not add up to 100% because of rounding.

^b^No cases of syphilis, gonorrhea, Hansen’s disease, or remission of addiction were found. All applicants 15 years of age or older must be tested for evidence of syphilis and gonorrhea; applicants younger than 15 must be tested if there is reason to suspect infection with syphilis or if there is a history of syphilis.

^c^Class B1: Have signs or symptoms, physical exam findings, or chest X-ray findings suggestive of tuberculosis disease, or have known human immunodeficiency virus infection, but have negative acid-fast bacilli (AFB) sputum smears and cultures and are not diagnosed with tuberculosis disease.

^d^Class B2: Positive interferon-γ release assay (IGRA) or TST but otherwise a negative evaluation for tuberculosis. Per CDC’s Tuberculosis Technical Instructions for Panel Physicians, applicants 2 to 14 years old living in countries with a World Health Organization–estimated tuberculosis incidence rate of ≥20 cases per 100,000 population must have an IGRA or, during the period of analysis, TST (tuberculosis incidence in 2017: Iraq—42 per 100,000 people; Afghanistan—189 per 100,000 people). An IGRA/TST is not required for applicants over the age of 14 years, so the denominator for the calculation of latent tuberculosis infection (LTBI) includes children between the ages of 2 and 14 only.

^e^Multivariable logistic regression models were fitted to estimate adjusted odds ratios (ORs) and 95% confidence intervals for health conditions where there were >10 counts per cell. If <10 counts per cell, the models and *p*-values were not calculated. Multivariable logistic regression models included the health condition as the outcome variable; SIVH origin (Iraqi or Afghan; Afghan as the reference) as the primary exposure variable; and age and sex as the covariates. A *p*-value of <0.05 was considered statistically significant.

^f^Abnormal vision findings included strabismus and congenital cataracts. Impairment in learning, intelligence, or communication findings included children identified with Down syndrome and cerebral palsy. Major mental disorders included personality disorder and mood disorders. Examples of visible disabilities could include visible loss of limbs, mobility limitations, and use of assistive devices.

Abbreviations: CDC, US Centers for Disease Control and Prevention; NA, Not Applicable; SIVH, Special Immigrant Visa holder; TST, tuberculin skin test

## Discussion

In this analysis of SIV children, less than 1% were reported to have abnormal tuberculosis test findings, less than 1% had hearing abnormalities, and about 4% had vision abnormalities, with a greater prevalence of vision abnormalities noted in Iraqis. Seizure disorders were noted in 0.3% children, with Iraqis more likely to have a seizure disorder. Compared to Afghan children <5 years of age, Iraqi children had greater observed mean weight-for-height and height-for-age z-scores. For children ≥5 years, Iraqi children similarly had greater observed mean BMI-for-age and height-for-age z-scores compared to Afghan children. Data quality assessment for height for age for children ≥5 years fell within WHO recommendations.

Few articles document latent tuberculosis infection (LTBI) in children from either country. One study reported an LTBI prevalence of 6.8% in Afghan unaccompanied minors in Sweden [8]. Although the estimate was higher than our finding of 0.1%, the authors of that study felt their rates could be related, in part, to exposure during transit. In contrast, our population primarily remained in Afghanistan before US resettlement. Furthermore, prior to October 2018, SIV children between the ages of 2 and 14 were required to receive TSTs overseas for their tuberculosis evaluation; since October 1, 2018, interferon-γ release assay (IGRA) testing is now required [9]. Due to the subjectivity of TST readings, LTBI may not have been identified among children with no other signs or symptoms of tuberculosis; however, the requirement of overseas IGRA testing may improve LTBI identification in the future. Additionally, this required change overseas may reduce both the time and financial burden required for LTBI follow-up conducted by state and local health departments [10–13].

There are few articles on chronic malnutrition among children 5 years or older. Studies of stunting in children under 5 years document a range from 40% to 60% for Afghanistan and from 7% to 23% for Iraq. It is possible that chronic malnutrition extends to older children if causes of chronic malnutrition, such as inadequate dietary intake or chronic illness, persist [14–16].

There are limitations to our analysis. First, not all SIV records are available, because the records were inconsistently entered into EDN. As such, our findings are not generalizable to all SIVH entering the US. Second, medical history for noncommunicable diseases was self-reported or reported by family. Because overseas physicians do not conduct a comprehensive medical examination for noncommunicable diseases, these conditions could be underdiagnosed, underreported, or both. Third, our logistic regression models did not take into account other factors potentially related to the health outcomes, such as socioeconomic status, access to health services, genetics, exposure to adverse events, and others. Lastly, given the presumed poor data quality of the anthropometric indicators, our findings should be interpreted with caution. Errors may be due to inaccuracy in age reporting or in measurement (e.g., rounding of anthropometric indicators at the time of measurement or from electronic systems housing the data). SDs for older children and Afghans were lower than for younger children and Iraqis, respectively, suggesting higher measurement quality in the former groups. CDC and international partners are conducting further investigation into the nature of the poor data quality.

Domestic providers caring for SIVH should follow CDC’s Guidelines for the US Domestic Medical Examination for Newly Arriving Refugees, including a thorough evaluation for LTBI, malnutrition, and overweight/obesity [17]. Effective measurement techniques and quality of anthropometric equipment used in panel site clinics should be assessed to be sure they can accurately determine the nutritional status of children. Additional training in measurement techniques should be considered. Future analyses could further explore health of SIV children after resettlement in the US.

## Supporting information

S1 STROBE Checklist (DOCX)

## Abbreviations

AFB: acid-fast bacilli
BMI: body mass index
CDC: US Centers for Disease Control and Prevention
EDN: Electronic Disease Notification system
IGRA: interferon-γ release assay
LTBI: latent tuberculosis infection
SD: standard deviation
SIVH: Special Immigrant Visa holder
TST: tuberculin skin test
WRAPS: Worldwide Refugee Admissions Processing System

## References

[1] Refugee Processing Center, Bureau of Population, Refugees, and Migration, Office of Admissions, US Department of State. Admissions and Arrivals [cited 2019 Mar 26]. Available from: https://www.wrapsnet.org/admissions-and-arrivals/.

[2] Division of Global Migration and Quarantine, Centers for Disease Control and Prevention, US Department of Health and Human Services. Medical Examination of Immigrants and Refugees. 2012 [cited 2019 Mar 26]. Available from: http://www.cdc.gov/immigrantrefugeehealth/exams/medical-examination.html.

[3] Division of Global Migration and Quarantine, Centers for Disease Control and Prevention, US Department of Health and Human Services. CDC Immigration Requirements: Technical Instructions for Other Physical or Mental Abnormality, Disease, or Disability. 2012 [cited 2020 Jan 12]. Available from: https://www.cdc.gov/immigrantrefugeehealth/exams/ti/panel/technical-instructions/panel-physicians/other-physical-mental.html.

[4] Lee D, Philen R, Wang Z, McSpadden P, Posey D, Ortega L, et al Disease surveillance among newly arriving refugees and immigrants—Electronic Disease Notification System, United States, 2009. Morbidity and Mortality Weekly Report: Surveillance Summaries. 2013; 62(7): 1–20. 24225411

[5] World Health Organization. World Health Organization Anthro for personal computers, version 3.2.2, 2011: Software for assessing growth and development of the world’s children. Geneva: World Health Organization; 2011 [cited 2019 Mar 26]. Available from: http://www.who.int/childgrowth/software/en/.

[6] World Health Organization. World Health Organization Growth Reference, 5–19 Years, BMI-for-age. Geneva: World Health Organization; 2007 [cited 2019 Mar 26]. Available from: https://www.who.int/growthref/who2007_bmi_for_age/en/.

[7] World Health Organization. World Health Organization Global Database on Child Growth and Malnutrition. Geneva: World Health Organization; 1997 [cited 2019 Mar 26]. Available from: https://www.who.int/nutgrowthdb/about/introduction/en/index5.html.

[8] Bennet R, Eriksson M. Tuberculosis infection and disease in the 2015 cohort of unaccompanied minors seeking asylum in Northern Stockholm, Sweden. Infectious Diseases. 2017; 49(7): 501–6. 10.1080/23744235.2017.1292540 28276801

[9] Division of Global Migration and Quarantine, Centers for Disease Control and Prevention, US Department of Health and Human Services. Tuberculosis Technical Instructions for Panel Physicians. 2019 [cited 2019 Apr 7]. Available from: https://www.cdc.gov/immigrantrefugeehealth/exams/ti/panel/tuberculosis-panel-technical-instructions.html.

[10] Schwartzman K, Oxlade O, Barr G, Grimard F, Acosta I, Baez J, et al Domestic returns from investment in the control of tuberculosis in other countries. New England Journal of Medicine. 2005; 353(10): 1008–20. 10.1056/NEJMsa043194 16148286

[11] Posey D, Naughton M, Willacy E, Russell M, Olson C, Godwin C, et al Implementation of new TB screening requirements for US-bound immigrants and refugees—2007–2014. Morbidity and Mortality Weekly Report. Morbidity and Mortality Weekly Report. 2014; 63(11): 234 24647399 PMC4584633

[12] Wingate L, Coleman M, Posey P, Zhou W, Olson C, Maskery B, et al Cost-effectiveness of screening and treating foreign-born students for tuberculosis before entering the United States. PLoS ONE. 2015; 10(4). 10.1371/journal.pone.0124116 PMC4414530 25924009

[13] Maskery B, Posey D, Coleman M, Asis R, Zhou W, Painter J, et al Economic analysis of CDC's culture-and smear-based tuberculosis instructions for Filipino immigrants. International Journal of Tuberculosis and Lung Disease. 2018; 22(4): 429–36. 10.5588/ijtld.17.0453 29562992 PMC6390485

[14] Afghanistan Ministry of Public Health, UNICEF. National Nutrition Survey Afghanistan (2013). 2013 [cited 2019 Mar 26]. Available from: https://reliefweb.int/report/afghanistan/national-nutrition-survey-afghanistan-2013 NNS Afghanistan 2013 (July 26–14).pdf.

[15] World Health Organization, Department of Nutrition for Health and Development. Nutrition Landscape Information System. Geneva: World Health Organization [cited 2019 Mar 26]. Available from: https://www.who.int/nutrition/nlis/en/.

[16] Dawson-Hahn E, Pak-Gorstein S, Hoopes A, Matheson J. Comparison of the nutritional status of overseas refugee children with low income children in Washington State. PLoS ONE. 2016; 11(1). 10.1371/journal.pone.0147854 PMC4725764 26808275

[17] Division of Global Migration and Quarantine, Centers for Disease Control and Prevention, US Department of Health and Human Services. Guidelines for the US Domestic Medical Examination for Newly Arriving Refugees. 2019 [cited 2019 Mar 26]. Available from: https://www.cdc.gov/immigrantrefugeehealth/guidelines/domestic/domestic-guidelines.html.

