# Supplementary material for: Health profile of pediatric Special Immigrant Visa holders arriving from Iraq and Afghanistan to the United States, 2009–2017: A cross-sectional analysis
Source: PLoS Med. 2020 Mar 17;17(3):e1003069. doi: 10.1371/journal.pmed.1003069 (PMC7077800; doi:10.1371/journal.pmed.1003069)
Supplement: S1 STROBE Checklist — (DOCX) [file pmed.1003069.s001.docx]

STROBE Statement—Checklist of items that should be included in reports of ***cross-sectional studies***

**Excerpts are listed below the checklist**

|  | Item No | Recommendation | Paragraph No |
| --- | --- | --- | --- |
| **Title and abstract** | 1 | (*a*) Indicate the study’s design with a commonly used term in the title or the abstract | See Title |
|  |  | (*b*) Provide in the abstract an informative and balanced summary of what was done and what was found | See Abstract |
| Introduction | | | |
| Background/rationale | 2 | Explain the scientific background and rationale for the investigation being reported | Introduction, paragraph 3 |
| Objectives | 3 | State specific objectives, including any prespecified hypotheses | Introduction, paragraph 3 |
| Methods | | | |
| Study design | 4 | Present key elements of study design early in the paper | Methods, paragraph 1 |
| Setting | 5 | Describe the setting, locations, and relevant dates, including periods of recruitment, exposure, follow-up, and data collection | Methods, paragraph 1 |
| Participants | 6 | (*a*) Give the eligibility criteria, and the sources and methods of selection of participants | Methods, paragraph 1 |
| Variables | 7 | Clearly define all outcomes, exposures, predictors, potential confounders, and effect modifiers. Give diagnostic criteria, if applicable | Methods, paragraph 2, Table 2 |
| Data sources/ measurement | 8* | For each variable of interest, give sources of data and details of methods of assessment (measurement). Describe comparability of assessment methods if there is more than one group | Methods, paragraph 1, 2, 3 |
| Bias | 9 | Describe any efforts to address potential sources of bias | N/A |
| Study size | 10 | Explain how the study size was arrived at | Methods, paragraph 1 |
| Quantitative variables | 11 | Explain how quantitative variables were handled in the analyses. If applicable, describe which groupings were chosen and why | Methods, paragraph 2, 3 |
| Statistical methods | 12 | (*a*) Describe all statistical methods, including those used to control for confounding | Methods, paragraph 3 |
|  |  | (*b*) Describe any methods used to examine subgroups and interactions | Methods, paragraph 3 |
|  |  | (*c*) Explain how missing data were addressed | Methods, paragraph 3 |
|  |  | (*d*) If applicable, describe analytical methods taking account of sampling strategy | N/A |
|  |  | (*e*) Describe any sensitivity analyses | N/A |
| Results | | | |
| Participants | 13* | (a) Report numbers of individuals at each stage of study—eg numbers potentially eligible, examined for eligibility, confirmed eligible, included in the study, completing follow-up, and analysed | Methods, paragraph 1 |
|  |  | (b) Give reasons for non-participation at each stage | N/A |
|  |  | (c) Consider use of a flow diagram | N/A |
| Descriptive data | 14* | (a) Give characteristics of study participants (eg demographic, clinical, social) and information on exposures and potential confounders | Table 1 |
|  |  | (b) Indicate number of participants with missing data for each variable of interest | Table 1, Table 2 |
| Outcome data | 15* | Report numbers of outcome events or summary measures | Table 1, Table 2 |
| Main results | 16 | (*a*) Give unadjusted estimates and, if applicable, confounder-adjusted estimates and their precision (eg, 95% confidence interval). Make clear which confounders were adjusted for and why they were included | Results, paragraph 1, 2 |
|  |  | (*b*) Report category boundaries when continuous variables were categorized | N/A |
|  |  | (*c*) If relevant, consider translating estimates of relative risk into absolute risk for a meaningful time period | N/A |
| Other analyses | 17 | Report other analyses done—eg analyses of subgroups and interactions, and sensitivity analyses | N/A |
| Discussion | | | |
| Key results | 18 | Summarise key results with reference to study objectives | Discussion, paragraph 1 |
| Limitations | 19 | Discuss limitations of the study, taking into account sources of potential bias or imprecision. Discuss both direction and magnitude of any potential bias | Discussion, paragraph 4 |
| Interpretation | 20 | Give a cautious overall interpretation of results considering objectives, limitations, multiplicity of analyses, results from similar studies, and other relevant evidence | Discussion, paragraphs 2, 3, 4 |
| Generalisability | 21 | Discuss the generalisability (external validity) of the study results | Discussion, paragraph 4 |
| Other information | | | |
| Funding | 22 | Give the source of funding and the role of the funders for the present study and, if applicable, for the original study on which the present article is based | N/A |

*Give information separately for exposed and unexposed groups.

**Note:** An Explanation and Elaboration article discusses each checklist item and gives methodological background and published examples of transparent reporting. The STROBE checklist is best used in conjunction with this article (freely available on the Web sites of PLoS Medicine at http://www.plosmedicine.org/, Annals of Internal Medicine at http://www.annals.org/, and Epidemiology at http://www.epidem.com/). Information on the STROBE Initiative is available at [www.strobe-statement.org](http://www.strobe-statement.org).

**Excerpts (per item number)**

1 A) “Health Profile of Pediatric Special Immigrant Visa Holders Arriving from Iraq and Afghanistan to the United States 2009–2017: a Cross-Sectional Analysis”

B) “We report the frequency of selected diseases identified overseas and assess differences in selected conditions between SIV children from Iraq and Afghanistan.”

See abstract

2 “Despite the increase in SIV admissions to the United States over recent years, little is known about health conditions in SIV children. Increasing provider knowledge regarding common health conditions in SIV children may facilitate improved care upon arrival to the United States.”

3 “We report the frequency of selected diseases identified overseas and assess differences in selected conditions between SIV children from Iraq and Afghanistan.”

4 “Iraqi and Afghan SIV children (<18 years) admitted to the United States from April 2009 through December 2017 were included. EDN and the US Department of State’s Worldwide Refugee Admissions Processing System (WRAPS) were used as data sources in this cross-sectional analysis [1, 4].”

5 “Iraqi and Afghan SIV children (<18 years) admitted to the United States from April 2009 through December 2017 were included. EDN and the US Department of State’s Worldwide Refugee Admissions Processing System (WRAPS) were used as data sources in this cross-sectional analysis [1, 4].”

6 “Iraqi and Afghan SIV children (<18 years) admitted to the United States from April 2009 through December 2017 were included. EDN and the US Department of State’s Worldwide Refugee Admissions Processing System (WRAPS) were used as data sources in this cross-sectional analysis [1, 4].”

7 “EDN variables included age, sex, measured height and weight, and results of the overseas medical examination while information about native language was obtained from WRAPS. Presence of non-communicable diseases was self-reported, and categorized as either “yes” or “no”.”

Other diagnostic criteria noted in Table 2.

8 “EDN and the US Department of State’s Worldwide Refugee Admissions Processing System (WRAPS) were used as data sources in this cross-sectional analysis [1, 4].”

“EDN variables included age, sex, measured height and weight, and results of the overseas medical examination while information about native language was obtained from WRAPS. Presence of non-communicable diseases was self-reported, and categorized as either “yes” or “no”.”

“This analysis was not guided by a specific prospective analysis plan. Frequencies were calculated to describe demographic characteristics and disease prevalence.”

10 “Iraqi and Afghan SIV children (<18 years) admitted to the United States from April 2009 through December 2017 were included. EDN and the US Department of State’s Worldwide Refugee Admissions Processing System (WRAPS) were used as data sources in this cross-sectional analysis [1, 4]. We identified 15,729 SIV children < 18 years of age.”

11 “This analysis was not guided by a specific prospective analysis plan. Frequencies were calculated to describe demographic characteristics and disease prevalence.”

“Mean z-scores and standard deviations (SD) were calculated for weight-for-height (if <5 years), body mass index (BMI)-for-age (if ≥5 years), and height-for-age (all ages) using the World Health Organization’s Statistical Analysis Software macros [5, 6].”

12 A) “This analysis was not guided by a specific prospective analysis plan Frequencies were calculated to describe demographic characteristics and disease prevalence. Mean z-scores and standard deviations (SD) were calculated for weight-for-height (if <5 years), body mass index (BMI)-for-age (if ≥5 years), and height-for-age (all ages) using the World Health Organization’s Statistical Analysis Software macros [5, 6]. Data quality of these anthropometric measurements was assessed using cut-off points for SD according to World Health Organization’s recommendations [7]. Multivariable logistic regression models adjusted for age and sex were used to assess associations of certain conditions with nationality. Health conditions with fewer than 10 cases were excluded from regression analyses. Denominators used to estimate the prevalence of medical conditions varied because of missing data. All statistical analyses were performed with SAS 9.3 (SAS Institute Inc., Cary, NC, USA).”

B) “Multivariable logistic regression models adjusted for age and sex were used to assess associations of certain conditions with nationality.”

C) “Denominators used to estimate the prevalence of medical conditions varied because of missing data.”

D) N/A

E) N/A

13 A) “Our analysis included 15,729 children (Table 1)”.

B) N/A

C) N/A

14 A) See Table 1

B) See Table 1 and Table 2

15 See Table 1 and Table 2

“Our analysis included 15,729 children (Table 1). For children <5 years of age, the observed mean weight-for-height z-score in Afghan children was lower than in Iraqi children (Iraqi +0.13; Afghan: -0.10), as was observed mean z-score for height-for-age (Iraqi: -1.09; Afghan: -1.37). However, the SDs for all anthropometric indicators were larger than suggested range for data quality (Table 1), suggesting potential inaccuracy in measurement or reporting [7].”

“For children ≥5 years, the mean height-for-age z-score in Afghan children was lower than in Iraqi children (Iraqi: -0.28; Afghan: -0.68), as was mean z-score for BMI-for-age (Iraqi: +0.25; Afghan: -0.41). However, the SDs for BMI-for-age were larger than expected (Table 1), suggesting potential inaccuracy in measurement or reporting [7].”

“Sixteen children had abnormal tuberculosis test findings: 3 (0.02%) had an abnormal chest x-ray with negative sputum cultures (Class B1) and, among those who received a tuberculin skin test (TST), 13 (0.1%) had a positive TST but negative chest x-ray (Class B2) (Table 2).”

“Sixteen children had abnormal tuberculosis test findings: 3 (0.02%) had an abnormal chest x-ray with negative sputum cultures (Class B1) and, among those who received a tuberculin skin test (TST), 13 (0.1%) had a positive TST but negative chest x-ray (Class B2) (Table 2). Vision abnormalities were noted in 566 (4%) children, with Iraqis twice as likely to have vision abnormalities (OR: 1.9, 95% CI: 1.6-2.2, *p* < 0.001). Among children reported with vision abnormalities, strabismus was reported for 92 children (0.5%). Hearing abnormalities were noted in 89 (0.6%) children, with Iraqis less likely to have hearing abnormalities (OR: 0.4, 95% CI: 0.2-0.7, *p* = 0.002). Seizure disorders were noted in 46 (0.3%) children, with Iraqis more likely to have a seizure disorder (OR: 7.6, 95% CI: 3.8-15.0, *p* < 0.001). No cases of syphilis, gonorrhea or Hansen’s disease were found.”

16 A) “Vision abnormalities were noted in 566 (4%) children, with Iraqis twice as likely to have vision abnormalities (OR: 1.9, 95% CI: 1.6-2.2, *p* < 0.001). Among children reported with vision abnormalities, strabismus was reported for 92 children (0.5%). Hearing abnormalities were noted in 89 (0.6%) children, with Iraqis less likely to have hearing abnormalities (OR: 0.4, 95% CI: 0.2-0.7, *p* = 0.002). Seizure disorders were noted in 46 (0.3%) children, with Iraqis more likely to have a seizure disorder (OR: 7.6, 95% CI: 3.8-15.0, *p* < 0.001). No cases of syphilis, gonorrhea or Hansen’s disease were found.”

B) N/A

C) N/A

17 N/A

18 “In this analysis of SIV children, less than 1% were reported to have abnormal tuberculosis test findings, less than 1% had hearing abnormalities, and about 4% had vision abnormalities, with a greater prevalence of vision abnormalities noted in Iraqis. Seizure disorders were noted in 46 (0.3%) children, with Iraqis more likely to have a seizure disorder (OR: 7.6, 95% CI: 3.8-15.0, *p* < 0.001). Compared to Afghan children <5 years of age, Iraqi children had greater observed mean z-scores for weight-for-height and height-for-age. For children ≥5 years, Iraqi children similarly had greater observed mean z-scores for BMI-for-age and height-for-age compared to Afghan children. Data quality assessment for height-for-age for children ≥5 years fell within WHO recommendations.”

19 “There are limitations to our analysis. First, not all SIV records are available, because the records were inconsistently entered into EDN. As such, our findings are not generalizable to all SIVH entering the US. Second, medical history for noncommunicable diseases was self-reported or reported by family. Because overseas physicians do not conduct a comprehensive medical examination for noncommunicable diseases, these conditions could be underdiagnosed, underreported, or both. Third, our logistic regression models did not take into account other factors potentially related to the health outcomes, such as socioeconomic status, access to health services, genetics, exposure to adverse events, and others. Lastly, given the presumed poor data quality of the anthropometric indicators, our findings should be interpreted with caution. Errors may be due to inaccuracy in age reporting or in measurement (e.g. rounding of anthropometric indicators at the time of measurement or from electronic systems housing the data). Standard deviations for older children and Afghans were lower than for younger children and Iraqis, respectively, suggesting higher measurement quality in the former groups. CDC and international partners are conducting further investigation into the nature of the poor data quality.”

20 “Few articles document latent tuberculosis infection (LTBI) in children from either country. One study reported an LTBI prevalence of 6.8% in Afghan unaccompanied minors in Sweden [8]. Although the estimate was higher than our finding of 0.1%, the authors of that study felt their rates could be related, in part, to exposure during transit. In contrast, our population primarily remained in Afghanistan before US resettlement. Further, prior to October 2018, SIV children between the ages of 2 through 14 were required to receive TSTs overseas for their tuberculosis evaluation; since October 1^st^ 2018, IGRA testing is now required [9]. Due to the subjectivity of TST readings, LTBI may not have been identified among children with no other signs or symptoms of tuberculosis; however, the requirement of overseas IGRA testing may improve LTBI identification in the future. Additionally, this required change overseas may reduce both the time and financial burden required for LTBI follow-up conducted by state and local health departments [10-13].”

“There are few articles on chronic malnutrition among children 5 years or older. Studies of stunting in children under 5 years document a range from 40% to 60% for Afghanistan, and from 7% to 23% for Iraq. It is possible that chronic malnutrition extends to older children if causes of chronic malnutrition, such as inadequate dietary intake or chronic illness, persist [14-16].”

See throughout the Discussion.

21 “First, not all SIV records are available, because the records were inconsistently entered into EDN. As such, our findings are not generalizable to all SIVH entering the US.”

22 N/A
